# Endometrial Signatures of Subfertility in Beef Heifers Reveal Dysregulation of MAPK Signaling and Ciliary Function

**DOI:** 10.3390/genes16111323

**Published:** 2025-11-03

**Authors:** Nicholas C. Kertz, Priyanka Banerjee, Paul W. Dyce, Soren P. Rodning, Wellison J. S. Diniz

**Affiliations:** Department of Animal Sciences, Auburn University, Auburn, AL 36849, USA; n.kertz@ufl.edu (N.C.K.); pzb0035@auburn.edu (P.B.); pwd0003@auburn.edu (P.W.D.); rodnisp@auburn.edu (S.P.R.)

**Keywords:** beef heifers, endometrium, gene expression, MAPK, subfertility, transcription factors

## Abstract

Background: Reproductive efficiency is a significant hurdle to the sustainability of the beef cattle industry. Method: This study employed transcriptomic profiling to investigate endometrial gene expression differences in heifers with divergent fertility outcomes. Caruncular endometrial samples from fertile (*n* = 7) and subfertile (*n* = 5) heifers were subjected to RNA-Seq analysis, yielding 894 differentially expressed genes (DEGs) (*p* ≤ 0.05 and |log2FC| ≥ 0.5). Results: The MAPK (Mitogen-activated protein kinase) and Rap1 (Ras-associated protein 1) signaling pathways and immune response regulation were identified among the over-represented pathways underlying the DEGs. Transcriptional regulators, such as *DUSP2*, *DUSP10*, and *MAPK13*, were downregulated in subfertile heifers, suggesting disrupted signal transduction and immune function. Gene co-expression network analysis showed network rewiring and increased connectivity of genes related to cilium organization, motility, and microtubule-based processes in the subfertile group. Over-represented hub genes were enriched in the subfertile endometrium, including *DNAH2*, *DNAI2*, *DNAAF4*, *CCDC65*, and the transcription factor *FOXJ1*. Our results suggest that impaired ciliary function and disrupted MAPK and immune signaling could potentially contribute to subfertility. Conclusions: This study highlights novel molecular signatures in the uterine endometrium that may serve as predictive markers of fertility potential in beef heifers, providing a foundation for targeted strategies to improve reproductive performance in cattle.

## 1. Introduction

Reproductive failure and pregnancy loss persist as the main challenges against herd productivity and selective management decisions [1]. Successful establishment and maintenance of pregnancy depend not only on embryo viability but also on a functionally competent and receptive uterine environment capable of supporting embryonic development [2,3]. A failure of the endometrium to establish an optimal environment before and during the implantation window is a leading cause of early embryonic loss and infertility [2,4]. Consequently, improving early pregnancy outcomes requires the identification of factors either indicative of a healthy and receptive endometrium or factors associated with a subfertile uterine environment.

Advancing our understanding of the physiological, molecular, and biochemical processes underpinning and regulating reproductive events is therefore a high priority. High-throughput sequencing technologies provide a framework for interrogating biological questions and enabling the discovery of novel biomarkers and pathways associated with reproductive success [5]. In particular, transcriptome profiling based on RNA-sequencing (RNA-Seq) has been used to characterize transcriptional changes in the bovine endometrium across stages of the estrous cycle and gestation [6]. Adhikari et al. identified 98 DEGs, including *MRS2*, *CST6*, *FOS*, *VLDLR*, *ISG15*, and *IFI6*, by comparing pregnant (day 15–17 of gestation) to non-pregnant heifers [7]. Other studies have shown that dynamic changes in gene expression within the intercaruncular endometrial regions are essential for pregnancy establishment [8,9,10]. Additionally, temporal differences in endometrial transcriptomic profiles across the estrous cycle (days 7, 14, and 15) [11,12,13,14,15,16] and early gestation [17] have been proposed as potential markers for reproductive potential in heifers.

Research efforts have increasingly focused on investigating the role of progesterone in modulating the uterine lumen and the gene transcription profiles within the uterine epithelium [18,19]. Similarly, attention has been given to the uterine luminal epithelial transcriptome to identify candidate genes that can predict pregnancy success [19,20,21]. Herein, we employed a transcriptomics-based analysis to identify DEGs in the caruncular endometrium of heifers with divergent fertility outcomes. We hypothesized that differences in the transcriptome profiles within the caruncular endometrium, mediated by transcription factors and regulatory networks, would alter underlying biological processes and pathways, contributing to varying reproductive outcomes. Therefore, we compared the endometrial transcriptome profiles of subfertile and fertile heifers to test this hypothesis. We identified 894 DEGs between the groups. Additionally, the connectivity profiles revealed specific co-expression patterns, characterized by a gain of connections in subfertile heifers. Notably, the caruncular endometrium of subfertile heifers exhibited significant transcriptional alterations, with enrichment of genes involved in MAPK signaling, immune response, and ciliary function.

## 2. Materials and Methods

### 2.1. Animal Management, Sample, and Heifer Classification

All procedures involving research animals were approved by the Institutional Animal Care and Use Committee (IACUC) at Auburn University (IACUC protocol number 2021-3968). Furthermore, experiments complied with the Planning Research and Experimental Procedures on Animals: Recommendations for Excellence (PREPARE) and Animal Research: Reporting of In Vivo Experiments (ARRIVE) guidelines.

Cross-bred (Simmental-Angus) heifers used in this study were sourced from two Auburn University research stations. Heifers’ pubertal status evaluation was performed approximately 120 days prior to the breeding season through reproductive tract scoring (RTS, scale of 1–5; 1 = pre-pubertal, infantile; 5 = pubertal, luteal phase) [22]. Immediately before the breeding season, heifers were subjected to an estrous synchronization for fixed-time artificial insemination (AI) utilizing the 7-Day CO-Synch + CIDR protocol. Heifers were synchronized with an intramuscular injection of 100 µg gonadotropin-releasing hormone (GnRH) (Cystorelin; Merial, Inc., Duluth, GA, USA) and an intravaginal progesterone insert (CIDR) providing 1.38 g of P4 (Eazi-Breed CIDR; Zoetis, Inc., Kalamazoo, MI, USA). After seven days and the removal of the intravaginal devices, heifers received an injection of prostaglandin F2α (Lutalyse, Zoetis Inc., Kalamazoo, MI, USA). Approximately 54 ± 2 h after CIDR removal, heifers were artificially inseminated with a single straw of semen from Angus sires and received a second intramuscular injection of 100 µg GnRH. Pregnancy status was determined 42 days post-AI via ultrasonography. Heifers that conceived to AI were deemed fertile, and seven were randomly selected for pregnancy termination with an injection of prostaglandin F2α, as we previously described [23]. All heifers that failed to conceive following AI (*n* = 20) underwent a 61-day period with fertile sires (*n* = 2) to allow sufficient opportunity for heifers to conceive. Pregnancy diagnosis was assessed using transrectal ultrasonography 13 and 34 days after the natural breeding season. Heifers that remained non-pregnant after AI and the 61-day breeding season (*n* = 5) were deemed subfertile and used in this study.

Fertility status was determined based on outcomes from AI and natural breeding. Heifers that became pregnant following the first AI were deemed fertile, whereas the ones remaining non-pregnant after AI and three estrous cycles with the bull were considered subfertile. All heifers (7 fertile and 5 subfertile) were consolidated and housed at the Auburn University teaching and research center, Auburn, AL. Heifers were confirmed cycling and harvested at the Auburn University Lambert-Powell Meats Lab on three different harvest dates following the final pregnancy check. The final body weight was measured for all the heifers before harvest. Uterine tracts were collected from heifers at harvest and transported to the lab on ice, where the collection of the uterine tract and ovarian measurements of each heifer was performed. Additionally, 0.1 g of the caruncle tissue was dissected from the midpoint of the right uterine horn. Sample tissues were kept in RNAlater (Invitrogen, Thermo Fisher Scientific, Waltham, MA, USA) at 4 °C for 24 h and then stored at −80 °C until further analysis.

### 2.2. RNA Isolation, Library Preparation, and RNA Sequencing

Total RNA was extracted from the endometrial tissue (30 mg) of 12 samples (7 fertile and 5 subfertile). The RNA was isolated following the TRIzol protocol (ThermoFisher Scientific Inc., Carlsberg, CA, USA). RNA purification was performed with DNase I (80 μL) (Qiagen, Germantown, MA, USA) followed by a 15 min incubation at room temperature. The RNA concentration and integrity quality (IQ) were verified using the Qubit RNA BR and Qubit RNA IQ Assay Kits on a Qubit Fluorometer v4.0 (Thermo Fisher Scientific Inc.). All samples had an IQ ≥ 7 and were subjected to directional mRNA library preparation (poly A enrichment) using NEBNext Ultra II Directional RNA Library Prep Kit for Illumina (New England BioLabs, Ipswich, MA, USA). Libraries were sequenced in the Illumina NovaSeq 6000 platform at Novogene Co. (Nanjing, China) to generate paired-end 150-bp reads at a depth of 20 million reads/sample.

### 2.3. RNA-Seq Data Analyses

We used FastQC v0.11.9 [24] to evaluate the sequencing quality and generate read statistics. FastQC output files were aggregated with MultiQC v1.12 [25], following our previously described pipeline [26]. Read mapping was performed with the STAR aligner v2.7.5 [27] to the Ensembl ARS-UCD 1.3 *Bos taurus* reference genome. Mapped read fragments were counted based on the *quantMode* gene counts option from the STAR aligner against the *B. taurus* annotation file from Ensembl (release 113).

Lowly expressed genes were removed using the *filterByExpr* function from edgeR v3.28.1 [28]. We implemented an ANOVA using the stats4 v3.6.3 R package to test for potential technical bias based on the variables collected during the breeding season and at harvest. An exploratory analysis was conducted using a principal component analysis (PCA) in the factoextra v1.0.7 [29]. The DEGs were identified using DESeq2 v1.26.1 [30] based on a multi-factor design (~location + pregnancy status) analysis. The DEGs with a *p* ≤ 0.05 and absolute log_2_ fold change (|log2FC|) ≥ 0.5 were considered significant. The DEGs were classified as up or downregulated based on the sign of the log2FC in the subfertile group. Gene annotation was performed using the BiomaRt v2.54.1 package [31]. The EnhancedVolcano v1.4.0 [32] was used to create a volcano plot to visualize the distribution of DEGs.

### 2.4. Identification of Key Transcription Factors (TFs)

Transcription factors modulate gene transcription by acting as molecular switches. Through the regulatory impact factor (RIF1 and RIF2) analysis, we predicted TFs differentially modulating gene transcription in the fertile and subfertile groups [33]. While the RIF1 provides TFs differentially connected to the DEGs, the RIF2 evaluates TFs potentially acting as predictors of DEGs’. To this end, the bovine TFs list (*n* = 1445) was retrieved from the Animal Transcription Factor Database (Animal TFDB v4.0) [34]. Next, TFs not present in our expression dataset were removed, retaining 1142 for further analysis. The RIF comparisons were performed on the fertile vs. subfertile groups. Significant TFs identified as potential regulators (RIF1 or RIF2) were selected considering a RIF score greater than |2.0| of the standard deviation (SD, *p* ≤ 0.05) [33].

### 2.5. Creating Gene Co-Expression Networks

Gene co-expression networks were created using the partial correlation coefficient and information theory algorithm [35]. Counts per million (CPM) were calculated from the raw counts using edgeR v3.28.1. The CPM normalized data were then used for network prediction (PCIT). Networks were created independently for fertile and subfertile heifers. The significant gene-gene correlations were considered with an absolute r ≥ 0.99, *p* ≤ 0.05. Additionally, gene pairs were retrieved when they included DEGs or TFs. Further, the Network Analyzer tool in Cytoscape v.3.8.0 was used to identify the hub genes. The degree measure from the network analyzer tool was used to identify the highly connected genes (hubs) by considering the Mean ± 2 SD. The differentially connected genes in each group were determined based on the differential connectivity (*DK*) score [36], as we previously described [37]. The $DK_{i}$ values were converted to *z*-scores, and those greater than ±1.96 SD were deemed significant (*p* ≤ 0.05).

### 2.6. Pathway Analysis

To assess the biological roles of the DEGs, an over-representation analysis was performed on ClueGO v2.5.10 [38]. For ClueGO, the genes were analyzed with *B. taurus* annotation as a background. The redundant terms were grouped based on the kappa score = 0.4 [38]. Significant KEGG pathways and biological processes terms were deemed significant (group *p*-value ≤ 0.05). A second approach was implemented using the gene set enrichment analysis (GSEA) [39]. To this end, all the expressed genes (*n* = 20,897) were ranked to detect sets of genes involved in shared pathways rather than focusing on individual DEGs. The ranks were calculated following the equation: rank =[sign (log 2 FC)×−log 10(p−value)] [40]. Based on that, GSEA assesses whether predefined gene sets associated with specific pathways or processes are enriched at the top or bottom of the ranked list [39]. The GSEA was performed using WebGestalt (WEB-based Gene SeT AnaLysis Toolkit) v.2024 [41]. This approach identifies over-represented KEGG pathways among genes ranked by expression values. The enrichment for each gene set is quantified by the normalized enrichment score (NES), which accounts for differences in gene set size and correlations with the expression data. A significant positive NES represents an enrichment of the gene set at the top of the ranked list (upregulated genes). In contrast, a significant negative NES reflects enrichment at the bottom (downregulated genes).

## 3. Results

### 3.1. Altered Expression of Genes and Transcription Factors in Subfertile Heifers

RNA-Seq analysis of the endometrium was used to identify differential expression and potential biological pathways underlying differences between fertile and subfertile heifers. The sequencing from all the samples yielded, on average, 32.28 million reads per sample, of which 31.01 million (96%) were uniquely mapped to the reference genome (Supplementary Table S1). After post-mapping QC, 20,897 out of 36,075 genes remained in 12 samples for further analysis. Normalized counts were analyzed by PCA. The clustering showed minimal separation between groups due to the fertility status (fertile or subfertile), and the first principal component explained 58.6% of the variability. Based on the ANOVA, no significant differences were observed between groups due to other covariates (harvest, RTS, weaning weight, final weight, reproductive tract length, and RNA IQs), except for the location of heifer origin (*p* = 0.01434). Therefore, location was included as a covariate in the DESeq2 model.

**Figure 1:**
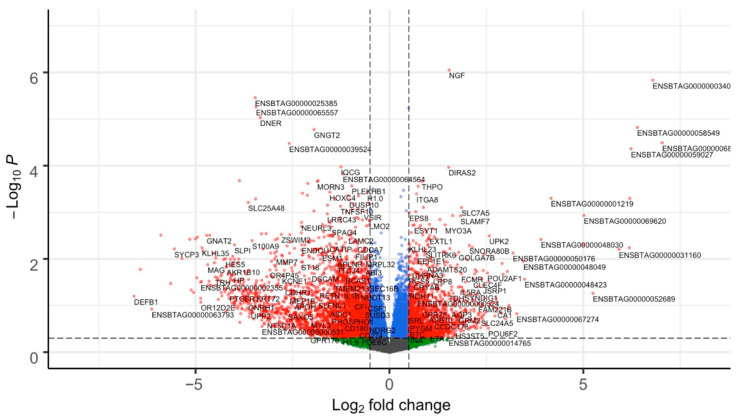
Volcano plot of differentially expressed genes (DEGs) from the endometrium tissue of subfertile and fertile heifers. Each dot represents a gene. The difference in gene expression between the fertile and subfertile groups is shown as the log2 fold change (*x*-axis). The −log (base 10) of the *p* is shown on the *y*-axis. Gene significance is color-coded as blue, green, and grey (non-significant genes that did not cross the threshold of *p* or fold-change); or red (significant 894 DEGs with *p* ≤ 0.05 and absolute (log2 fold change ≥ 0.5)). The genes were classified as up or downregulated based on the sign of the log2 fold change in the subfertile group. The significance threshold (*p* ≤ 0.05) is represented by the horizontal dashed line, while the fold change cutoff for up and downregulated genes (|log2FC| ≥ 0.5) is represented by the vertical dashed lines.

From the differential expression analysis in DESeq2, 894 DEGs were identified between subfertile and fertile groups (*p* ≤ 0.05 and |log2FC| ≥ 0.5) (Figure 1). Among these, 226 DEGs were upregulated and 668 DEGs were downregulated in the subfertile heifers (Supplementary Table S2). Although the majority of DEGs were protein-coding genes, long non-coding and small RNA-coding genes were also identified (Supplementary Figure S2). From the RIF metrics, we identified 104 unique TFs as potential regulators of the DEGs (*p* ≤ 0.05; RIF1 = 63; RIF2 = 52) out of 1142 tested (Supplementary Table S3).

### 3.2. Network Rewiring and Gain of Connectivity in the Subfertile Group

The co-expression networks between the subfertile and fertile groups were constructed using the PCIT algorithm. The co-expression of 20,897 genes resulted in 5,867,264 significantly co-expressed pairs in the fertile group, while there were 1,902,930 correlations for the subfertile group. The correlations were further filtered with absolute r > 0.99, DEGs, and TFs for data dimension reduction. After filtering, 36,867 and 1217 significantly correlated pairs were retrieved for the subfertile and fertile groups, respectively (Supplementary Table S4). These significantly correlated gene pairs were used for the dynamic network analysis to visualize the most rewired nodes across the network from the subfertile and fertile groups. From the gene network analysis in Cytoscape, the genes with the highest degree (highly connected to other significant genes) were identified as hubs. We identified 215 and 47 genes as hubs in the subfertile and fertile groups, respectively (Supplementary Table S5). The top hubs for the fertile group included *ADRA2C*, *ITGA8*, *RARA*, *PPP1R14D,* and *HOXB3*, while for the subfertile group included *SPMIP4*, *ENSBTAG00000064624*, *ENSBTAG00000069371*, *AOX4*, and *ENSBTAG00000066429*. After overlapping the 47 and 215 hub genes, 199 genes were exclusive to the subfertile group, while 31 genes were for the fertile group (Supplementary Table S5). The central reference network for fertile and subfertile groups was constructed using DyNet from 8678 nodes (genes) and 38,050 edges (interactions) (Supplementary Table S6). The *DK* analysis identified 206 differentially connected genes, of which 181 genes gained new connections in the subfertile group. Interestingly, *FOXJ1*, *ZNF892*, and *ENSBTAG00000019881* were differentially connected with greater connectivity in the subfertile network group and identified as significant DEG and TF.

### 3.3. Genes Underlying MAPK, RAP1 Signaling, and Immune Response Pathways Are Over-Represented

**Figure 2:**
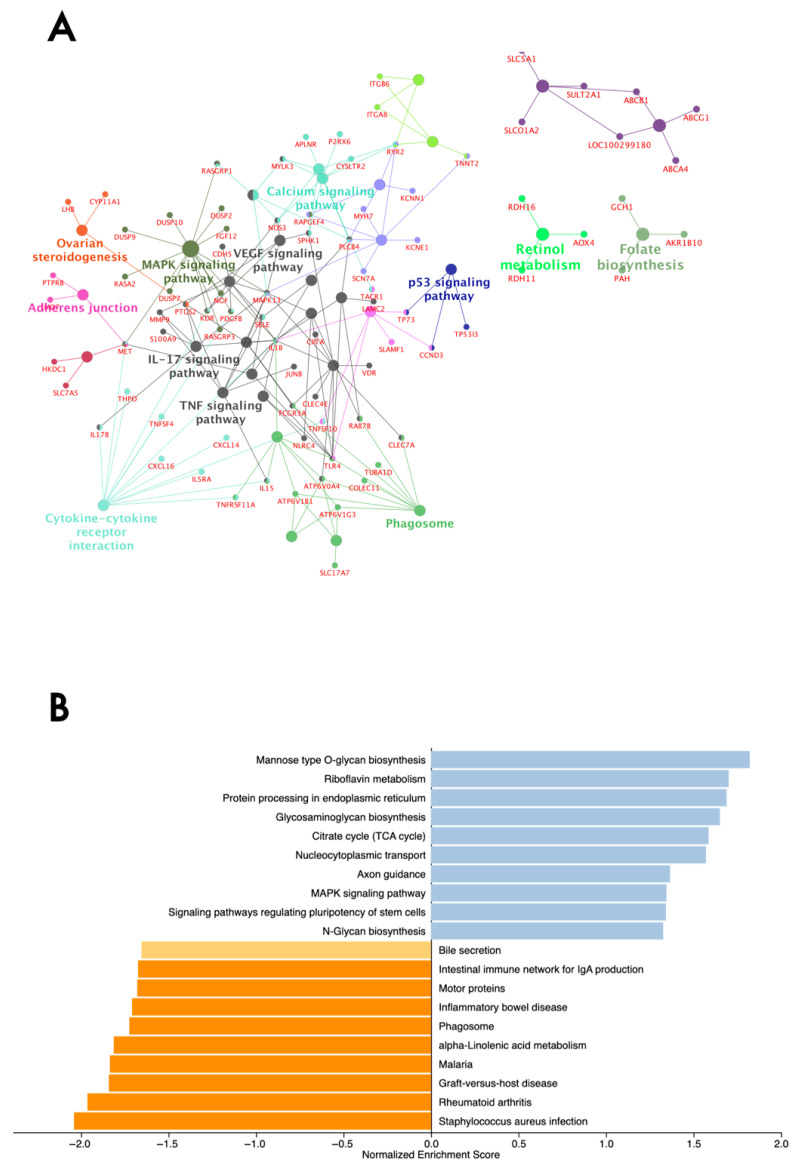
Over-representation analysis of differentially expressed genes from the endometrium tissue of subfertile and fertile beef heifers. (**A**) Over-represented KEGG pathways and genes identified through ClueGO. Functionally related terms partially overlap and are randomly colored. The significance of pathway enrichment is represented as the node size. (**B**) Gene set enrichment analysis of expressed genes. The normalized enrichment score (NES) of top-enriched (blue bars) and top-depleted (orange bars) pathways is based on the comparison of subfertile and fertile heifers. Only pathways with an NES ≥ |1.0| are shown.

We used two approaches to retrieve biological insights from the DEGs and hub genes. First, the pathway enrichment analysis was performed using ClueGO v2.5.10 based on the DEG list. The top significant KEGG pathways over-represented by DEGs included TNF, VEGF, IL-17, MAPK, and Rap1 signaling pathways. Other pathways included ovarian steroidogenesis, retinol metabolism, and folate biosynthesis (Supplementary Table S7a). Genes encoding dual-specificity phosphatases (DUSP), particularly *DUSP10*, *DUSP2*, *DUSP7*, and *DUSP9*, *MET*, *NGF*, *PDGFB*, *MAPK13*, and the Ras family of genes, including *RASA2*, *RASGRP1*, *RASGRP3,* were involved with the MAPK signaling pathway (group *p*-value ≤ 0.05). The DEGs underlying immune system pathways included *MAPK13*, *MMP9*, and *PTGS2* in the TNF and IL-17 signaling pathways; *IL15* in the TNF and cytokine-cytokine receptor interaction pathways; and *IL1B*, which was involved in all three pathways. The over-represented KEGG pathways and genes are given in Figure 2A.

Second, a GSEA was performed to provide a complete view of pathways, ranking all expressed genes (*n* = 20,897) considering a combination of *p* and log2FC. The top 20 KEGG pathways were identified from the normalized enrichment score (|NES > 1|) (Figure 2B). The top pathways over-represented were riboflavin metabolism, glycosaminoglycan biosynthesis, and the MAPK signaling pathway, which included *RASA2*, *DUSP3*, *DUSP4*, *DUSP8*, *DUSP9*, *VEGFD*, and *MAPK* genes. Inflammation and infection-related pathways were over-represented by genes with a negative ranking, suggesting a potential downregulation. Among the genes were included the BOLA-DRB family (*BOLA-DRB2*, *BOLA-DMB*, *BOLA-DRB3*, *BOLA-DMA*), interleukins (*IL15*, *IL6*, *IL10*, *IL1B*, and *IL18*), tumor necrosis factor (*TNFSF11A*, *TNFSF11*, and *TNFSF13B*), and *MAP3K14*.

**Figure 3:**
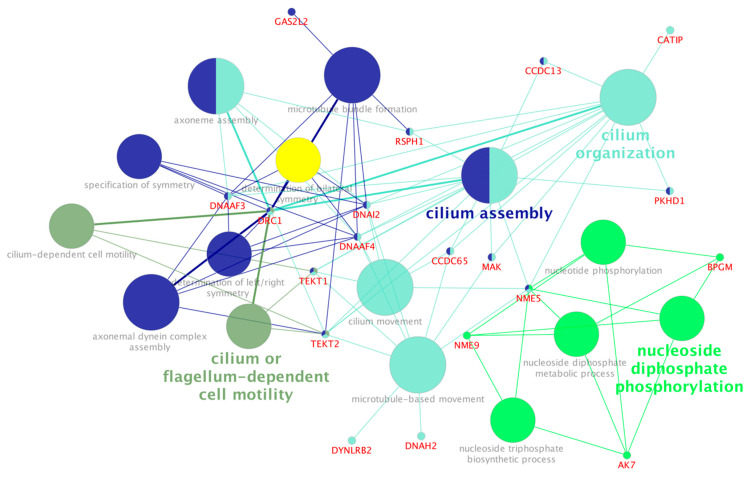
KEGG pathway over-representation analysis of hub genes identified from the expression profile of the endometrium tissue of subfertile beef heifers. Functionally related terms partially overlap and are randomly colored. The significance of pathway enrichment is represented as the node size.

The exclusive hub genes for the subfertile group (199 genes) were over-represented for biological processes including nucleoside phosphorylation, cilium or flagellum-dependent cell motility, microtubule bundle formation, and axonemal dynein complex assembly (Supplementary Table S7b, Figure 3). Some hub genes identified in the subfertile group, including members of the CCDC family (*CCDC65* and *CCDC13*), DNA family (*DNAAF4*, *DNAI2*, and *DNAH2*), TEKT family (*TEKT1* and *TEKT2*), as well as *MAPK* and *CATIP,* were significantly over-represented in pathways related to cilium organization, movement, and assembly (Figure 3).

## 4. Discussion

Successful establishment of pregnancy requires a tight and coordinated molecular crosstalk between the embryo and the maternal endometrium. Additionally, embryonic viability and the uterine environment are key determinants of early pregnancy success [17]. Similarly, dysregulation of multiple endometrial genes and pathways may lead to implantation failure and pregnancy loss [42]. Herein, we used the RNA-Seq approach to identify DEGs and underlying pathways involved with endometrial function in heifers with varying reproductive outcomes. We have identified 894 DEGs between subfertile and fertile heifers, underlying key pathways and biological processes involved with cell proliferation and function, and immune regulation.

We reported differences in the network topology between groups. The connectivity profiles revealed specific co-expression patterns, with a network connectivity gain in the subfertile heifer group. The DEGs were rewired, and eight out of 15 differentially connected TFs identified as key regulators (RIF) were more connected in the subfertile group. This rewiring involves changes in gene-gene interaction, creating new co-expression patterns that may be linked to adaptive response [43], or, in our case, the pregnancy outcome. Among the rewired TFs were identified *HOXB2* and *HOXB3*, which are part of the *HOX* family. As TFs, *HOX* genes have been associated with the modulation of target genes involved in endometrial development and receptivity [44]. From the interferon regulatory factor family, the *IRF8* TF also gained connectivity in the subfertile group. Genes from this family are induced by interferons, playing key roles in the immune system [45]. The *IFIT2* and *PTGS2* genes are interferon-stimulated and were downregulated in the subfertile heifers. *PTGS2* has several functions in the endometrium, indicating a receptive uterine environment. Therefore, the downregulation and rewiring of these genes in the endometrium of the subfertile group may be associated with failure in pregnancy recognition due to an environment that is unreceptive.

### 4.1. MAPK and Immune Signaling Pathways Are Over-Represented by Differentially Expressed Genes Between Subfertile and Fertile Classified Heifers

We identified significant genes encoding dual-specificity phosphatases (DUSP), particularly *DUSP10*, *DUSP2*, *DUSP7,* and *DUSP9*, within the MAPK signaling pathway. Notably, *DUSP2* was downregulated and identified as a hub gene in the subfertile group. This finding aligns with previous studies in humans, where *DUSP2* was downregulated in stromal cells of endometriotic tissues [46]. *DUSP2* plays a critical role in cell death and inflammation [47,48], and its activity is primarily associated with activated immune effector cells, including T cells, B cells, macrophages, and mast cells [49]. Similarly, *DUSP10* was downregulated in subfertile heifers. In our previous work, *DUSP10* was identified in the peripheral white blood cells of subfertile heifers and correlated with the bta-miR-92b in the subfertile group [50]. A key feature of the DUSP family is the ability to dephosphorylate signaling molecules, including MAPKs, thereby modulating the duration, intensity, and spatial distribution of MAPK signaling activity [51].

MAPK signaling regulates epithelial and stromal cell proliferation during the proliferative phase in humans. Additionally, it mediates progesterone-induced decidualization of stromal cells during the secretory phase, both of which are critical for establishing a receptive environment for embryo implantation [52]. Moreover, the MAPK pathway regulates immune tolerance and inflammatory responses necessary for successful embryo attachment and invasion [53]. Dysregulation of MAPK signaling has been associated with impaired endometrial receptivity, implantation failure, and infertility, emphasizing its role in female reproductive success [54]. Dickson et al. [55] speculate that *DUSP1* may influence the establishment and maintenance of pregnancy in cattle by dysregulating MAPK signaling. Furthermore, the MEK/ERK signaling cascade, a key component of the MAPK pathway, plays a potential role in female fertility [56]. Interestingly, we identified *BARX1* as a potential TF regulator of DEGs. Similarly, others have reported that depletion of *BARX1* is associated with the inactivation of the ERK/MEK signaling pathway [57]. Inactivation of the ERK/MEK signaling pathway in female mammals can lead to infertility by disrupting several key processes in reproduction, including oocyte maturation, ovulation, and early embryonic development [58]. Sigdel et al. [59], studying pregnancy loss in Holstein cows, reported that the MAPK and Rap1 pathways were over-represented by genes identified through a whole-genome scan.

The Rap1 pathway has been associated with basic cellular functions and uterine decidualization in rats [60]. Likewise, it plays a role in vascular stability during mouse embryonic development [61]. The Rap1 signaling pathway was significantly over-represented among DEGs in our study, involving genes such as *FGF12*, *MAPK13*, *NGF*, *PDGFB*, *PLCB4*, *RAPGEF4*, and *RASGRP3*. Except for *FGF12* and *NGF*, all these genes were downregulated in the subfertile heifer group. *PDGFB*, a growth factor essential for normal prenatal development [62], was previously reported to be upregulated in the corpus luteum of pregnant cows [63] and in the placenta of mice [62]. In contrast, reduced *PDGFB* expression has been observed in endometrial endothelial cells of women with abnormal uterine bleeding linked to endometrial disorders [64]. Furthermore, the *RASGRP3* gene regulates cell growth, differentiation, and survival [65]. *MAPK13* was identified as a common component across multiple pathways, including Rap1 signaling, MAPK signaling, VEGF signaling, and IL-17 signaling, underscoring its potential role in the molecular mechanisms underlying subfertility. *MAPK13* regulates cellular proliferation, differentiation, transcription, and development, and is activated by inflammatory cytokines and stress stimuli [66]. In humans, *MAPK13* has a potential role in the regulation of gonadotropins [67] and gynecological cancer [68]. Furthermore, it has been reported to be significantly upregulated in the endometrial luminal epithelium of pregnant mares [69], reinforcing its relevance in reproductive processes.

The immune signaling pathways, such as TNF, IL-17, and cytokine-cytokine receptor interaction, were over-represented by DEGs, including those from the interleukin family (*IL15*, *IL1B*, *IL17B*, *IL5*, and *IL5RA*), chemokines (*CXCL12* and *CXCL16*), tumor necrosis factor (*TNFRSF11A*, *TNFSF10*, and *TNFSF4*), and *MMP9*, *PDGFB*, *KDR,* and *MET* genes. Inflammation and immune response have been associated with fertility and reproductive outcomes in cattle [70]. We have previously reported a downregulation of immune-related genes in the peripheral white blood cells of subfertile heifers at weaning. Similarly, Rocha et al. [20] identified genes from endometrial epithelial cells that were negatively associated with pregnancy success in Brahman cows, including those involved with chemokine and interleukin signaling. Maternal cytokines and chemokines play a key role in endometrial physiology and pregnancy recognition [71]. In the early stages of gestation, the maternal immune system is responsible for accepting or rejecting the embryo during implantation [3,72]. The interleukin-1 (IL-1) cytokine and receptor family relationship with blastocyst-endometrium signaling has been well investigated in bovines [73,74,75]. Furthermore, high levels of IL-1B were positively associated with implantation after in vitro fertilization in humans [76]. In our study, we found the *IL1B* and *IL17B* genes downregulated in subfertile heifers.

Additionally, we identified *MMP9* as significantly downregulated in subfertile heifers. Matrix metalloproteinases (MMPs) play an important role in modulating the vascular and uterine remodeling. Studies have indicated increased *MMP9* with vasodilation, placentation, and uterine expansion during a normal pregnancy in humans [77]. Decreased vascular *MMP9* expression may lead to reduced vasodilation, increased vasoconstriction, hypertensive disorders of pregnancy, and preeclampsia in humans [77]. Silva et al. [18] observed a downregulation of the *MMP1*, *MMP3*, and *MMP9* genes in the network underlying the extracellular matrix remodeling at day 7 post-estrus on the luminal transcriptome of cross-bred cows. Interestingly, the expression of *MMP9*, *MMP12*, and *MMP25* genes in the uterine epithelial cells four days after estrus was negatively associated with pregnancy in beef cows [20]. These supporting studies and functions in different species potentially warrant further investigation and validation of MMP genes in heifers with varying reproductive outcomes.

### 4.2. Hub Genes in the Subfertile Group Were Over-Represented for Microtubule-Based Movement, Cilium Movement and Assembly, and Axoneme Assembly

Among the hub genes identified in the subfertile group, the CCDC family (*CCDC65* and *CCDC13*), the DNA family (*DNAAF4*, *DNAI2*, and *DNAH2*), TEKT family (*TEKT1* and *TEKT2*), as well as the *MAK* and *CATIP* genes, were all downregulated and significantly over-represented in biological process terms related to cilium organization, movement, and assembly. Motile cilia are essential for human development and physiological homeostasis, and structural and functional defects are linked to a growing number of ciliopathies (cilia-related diseases). These include abnormalities in left-right body patterning during embryogenesis [78], infertility [79], and hydrocephalus [80]. In the endometrium, motile cilia play a critical role in embryo implantation and maintaining a receptive uterine environment [81]. The cilia have been involved with cellular signal transduction [82]. Impaired ciliary function may negatively affect the uterine epithelium’s ability to detect and respond to embryonic signals, leading to implantation failure [83]. Furthermore, ciliated epithelial cells may influence the immune microenvironment [84], which may adversely affect the immune tolerance needed for embryo implantation.

Histological abnormalities in cilia on the endometrial epithelium have been associated with recurrent pregnancy loss in women [81]. Impaired or absent ciliary motility can result in primary ciliary dyskinesia (PCD), a condition that causes female infertility [85]. PCD has been associated with homozygous mutations in *DNAAF4* [86], as well as mutations in *DNAI2* [87] and *DRC1* [88]. Interestingly, all these genes were identified as hubs in the subfertile group. In cattle, the higher levels of expression of *CCDC13*, *CCDC65*, and *AK7* genes in healthy cows compared to those with uterine disease [89] is consistent with their expression in subfertile heifers in our study. Mutations in *FOXJ1*, a key transcriptional regulator of motile ciliogenesis, have also been implicated in ciliary dysfunction [87]. Notably, we identified *FOXJ1* as a differentially connected TF that gained connectivity in the subfertile network. Although ciliary function in the endometrium has been reported in some studies [16,90], research specifically addressing its role in subfertility among cattle, specifically beef heifers, remains limited. These findings underscore the importance of further investigation into endometrial cilia and their potential contribution to fertility outcomes in cattle.

We identified novel genes and pathways differentially expressed in the endometrium of heifers with varying reproductive potential. One of the limitations, however, is the sample size (7 fertile and 5 sub-fertile). Therefore, confirming these targets in a larger cohort is necessary to confirm their relevance and assess their potential benefits to the beef cattle industry. The second limitation is that the endometrium comprises different cell compartments, such as luminal epithelial, glandular epithelial, and stromal cells, which are subjected to intense tissue remodeling during the estrous cycle, embryo implantation, and puerperal involution [91]. While this cellular complexity results in an averaged signal across cell populations, it does not capture cell type–specific responses, potentially limiting the interpretation of gene expression patterns and the functional roles of individual cell types [92]. However, the observed transcriptional differences likely reflect biologically meaningful responses rather than artifacts of tissue heterogeneity. Thus, evaluating the transcriptional patterns in different cell types would further highlight the interactions between each cell compartment that lead to heifers with varying reproductive potential. Further investigation, including multi-tissue analyses, could shed light on the relationships between the enriched pathways and the DEGs. Likewise, a longitudinal evaluation of these genes could better elucidate the relationship between immune function and early pregnancy success.

## 5. Conclusions

Our findings demonstrate that subfertility in beef heifers is associated with significant transcriptional changes in the caruncular endometrium, including downregulation of MAPK and immune signaling genes, as well as those underlying ciliary function. Identifying key regulators such as *DUSP2*, *MAPK13*, and *FOXJ1*, along with hub genes involved in cilia motility and assembly, suggests that dysregulation of signal transduction and structural defects in cilia may be associated with failure in pregnancy recognition. These results highlight the importance of endometrial receptivity in fertility outcomes and support using transcriptomics to identify molecular biomarkers predictive of reproductive potential. Further investigation is warranted to validate these results in larger populations and to assess the impact of candidate genes on endometrial physiology and fertility.

## Data Availability

All relevant data are included in the paper and its Supplementary Materials. All sequencing data is deposited on the GEO database.

## Supplementary Materials

The following supporting information can be downloaded at https://www.mdpi.com/article/10.3390/genes16111323/s1, Supplementary Table S1: Summary and mapping statistics of RNA-Sequencing data from the endometrium tissue of fertile and subfertile beef heifers; Supplementary Table S2: Differentially expressed genes in the endometrium tissue of beef heifers classified as fertile or subfertile. The up or downregulated genes were based on subfertile heifers (*p* ≤ 0.05 and |log2 FC| ≥ 0.5); Supplementary Table S3: Significant transcription factors (104 TFs) identified in the endometrium tissue of fertile and subfertile beef heifers. Regulatory TFs were identified based on the Regulatory Impact Factor (RIF1 and RIF2) scores. TFs with RIF1 and RIF2 z-scores ± 2 standard deviations were considered significant (*p* ≤ 0.05); Supplementary Table S4: Co-expressed gene pairs with differentially expressed genes and transcription factors identified with the PCIT approach; Supplementary Table S5: Network analysis of genes co-expressed with differentially expressed genes in fertile and subfertile groups. The hub genes for each group are highlighted in bold. The hub genes exclusive to each group are highlighted in bold and italics; Supplementary Table S6: Central reference network including fertile and subfertile networks constructed using 8678 nodes. The genes highlighted in bold have a z-score of ±1.96; the connectivity is given as gain or loss in the subfertile group; Supplementary Table S7: Functional over-representation analysis of differentially expressed genes in the endometrium tissue of beef heifers classified as fertile or subfertile.

## Abbreviations

The following abbreviations are used in this manuscript:

AI	Artificial insemination
BP	Biological processes
CIDR	Controlled internal releasing device
DEGs	Differentially expressed genes
DK	Differential Connectivity
FC	Fold Change
GnRH	Gonadotropin-releasing hormone
GSEA	Gene Set Enrichment Analysis
IQ	Integrity quality
KEGG	Kyoto encyclopedia of genes and genomes
log2FC	log 2-fold change
IACUC	Institutional Animal Care and Use Committee
K	Connectivity
MAPK	Mitogen-activated protein kinase
NES	Normalized Enrichment Score
P4	Progesterone
PCA	Principal component analysis
PCD	Primary ciliary dyskinesia
PCIT	Partial correlation and information theory
QC	Quality control
Rap1	Ras-associated protein 1
RIF	Regulatory impact factor
RNA-Seq	RNA sequencing
RTS	Reproductive tract score
SD	Standard deviation
TF	Transcription Factors
